# Serum Inflammatory Markers and Dietary Inflammatory Index in Obese Individuals With Fatty Liver Disease: A Case–Control Study

**DOI:** 10.1002/edm2.70181

**Published:** 2026-03-03

**Authors:** Faezeh Tejareh, Laleh Payahoo, Seyed Ali Keshavarz

**Affiliations:** ^1^ Department of Nutrition, Science and Research Branch Islamic Azad University Tehran Iran; ^2^ Medicinal Plants Research Center Maragheh University of Medical Sciences Maragheh Iran; ^3^ Department of Clinical Nutrition, School of Nutritional Sciences and Dietetics Tehran University of Medical Sciences Tehran Iran

**Keywords:** C‐reactive protein, dietary inflammatory index, fatty liver disease, inflammation, obesity

## Abstract

**Background:**

Fatty liver disease is common among obese individuals and is closely linked to chronic low‐grade inflammation. This study examines the relationship between dietary inflammatory index (DII) and serum inflammatory markers in obese individuals with and without non‐alcoholic fatty liver disease (NAFLD).

**Patients and Methods:**

In this case–control study, 85 obese adults (BMI ≥ 30), aged 20–70 years, were recruited from two outpatient clinics in Urmia, Iran. NAFLD diagnosis was based on sonography by an endocrinologist. Serum C‐reactive protein (CRP) levels were measured using a high‐sensitivity immunoturbidimetric assay to assess systemic inflammation. Dietary intake was assessed using a validated semi‐quantitative food frequency questionnaire (FFQ). The Dietary Inflammatory Index (DII) was calculated to evaluate the inflammatory potential of the diet.

**Results:**

Serum CRP levels were significantly higher in the NAFLD group (12.2 mg/L; 95% CI: 9.7–14.7) than controls (8.5 mg/L; 95% CI: 6.0–11.0; *p* = 0.04). DII scores did not differ significantly between groups (*p* = 0.2). CRP was positively associated with NAFLD (OR = 2.89; 95% CI: 1.1–7.2), while DII showed no significant association (OR = 0.50; 95% CI: 0.2–1.2).

**Conclusion:**

Our findings underscore the role of inflammation in the pathophysiology of fatty liver disease among obese individuals. Elevated CRP levels highlight potential targets for intervention. Although no significant differences in DII were observed, further serum investigation into the relationship between diet, inflammation and fatty liver is warranted.

## Graphical abstract

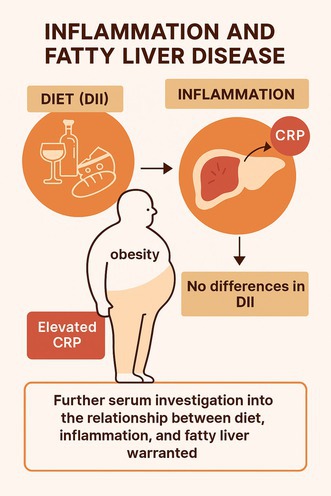

## 1 Introduction

Fatty liver (FL) disease is defined by the accumulation of fat exceeding 5%–10% of the liver's total weight [1]. This disease is a broad spectrum of mild liver disease in the form of fat accumulation in liver cells (steatosis), which, if not properly controlled, will develop into nonalcoholic steatohepatitis and ultimately into chronic and irreversible cirrhosis [2]. The prevalence of FL in adults in Western societies has been reported to be 34%–46%, which reaches 70%–80% in obese individuals [3]. In Iran, the prevalence of the disease was reported to be 36.9% in adults and 6.7% in children [4]. The high prevalence and chronic nature of this disease affect individuals' quality of life and impose a substantial economic burden on society, underscoring the need for effective health policies aimed at early identification and prevention [5]. It requires more than ever to identify modifiable risk factors and take measures to address them.

In most cases, FL is asymptomatic and can be diagnosed by observing elevated liver enzymes in a blood test or abdominal ultrasound; although some patients complain of vague pain in the upper right abdomen or a feeling of premature fatigue [6]. A wide range of factors can influence the aetiology of the disease, including metabolic disorders such as obesity, hyperlipidemia, diabetes, oxidative stress and prolonged starvation. Other important risk factors affecting the occurrence of fatty liver include an unhealthy lifestyle, which is characterised by poor nutritional status, low physical activity and environmental stress [7].

According to previous studies, dietary patterns can influence the risk of developing fatty liver disease through their nutrient composition and inflammatory potential. For example, western diets with high calorie content, high salt and sugar content, and high fat content, which are poor in terms of antioxidants and nutrients, aggravate the incidence of FL [8], while Mediterranean diets have shown protective effects on FL [9]. One of the most important factors in the development of fatty liver disease is an imbalance in inflammatory and anti‐inflammatory stimuli [10]. Inflammation associated with steatosis arises from the activation of immune cells and the subsequent secretion of pro‐inflammatory cytokines [11]. In fact, increased lipolysis of adipose tissue, high dietary fat intake and high levels of circulating fatty acids increase hepatic and systemic inflammation [10]. Interestingly, inflammation was reported to initiate a vicious cycle between obesity and nonalcoholic fatty liver disease [10].

Given the growing prevalence of fatty liver disease (FL) in the Iranian population—including both adults and children—and the lack of studies simultaneously examining serum and dietary inflammatory markers in obese individuals with and without FL, this case–control study aimed to compare serum C‐reactive protein (CRP) levels and Dietary Inflammatory Index (DII) scores between obese patients with NAFLD and healthy obese controls. The objective was to assess whether systemic and dietary inflammation are associated with the presence of fatty liver disease in this population. We hypothesised that individuals with fatty liver disease would exhibit higher levels of systemic inflammation, as measured by serum CRP, and a more pro‐inflammatory dietary pattern, as reflected by the Dietary Inflammatory Index (DII), compared to healthy obese individuals.

## 2 Methods

In this case–control study, a total of 85 overweight individuals, with or without fatty liver disease, were recruited through convenience sampling from patients referred to Taleghani Hospital and Tadbir Clinic in Urmia, Iran. The sample size was calculated using the formula for comparing two means in case–control studies [12]. Parameters included a 95% confidence level (*α* = 0.05), 90% power (*β* = 0.10), and an estimated standard deviation derived from prior literature. An attrition rate of 15% was also considered. The calculation followed standard epidemiological methods as described by Charan and Biswas [13]. Inclusion criteria comprised an age range of 20–70 years and a body mass index (BMI) greater than 30 kg/m^2^. Obesity was defined as BMI ≥ 30 kg/m^2^ according to the general WHO criteria. However, to account for potential ethnic differences, a sensitivity analysis was also performed using the Asian‐specific cut‐off of BMI ≥ 27.5 kg/m^2^ to ensure robustness of the findings. NAFLD diagnosis was based on hepatic ultrasound performed by a board‐certified endocrinologist using standardised criteria for steatosis, including increased echogenicity and poor visualisation of intrahepatic vessels. The control group consisted of healthy overweight and obese individuals who were referred to the same hospital and had no familial relationship with the affected patients. To minimise potential selection bias, both cases and controls were recruited from the same clinical settings and matched on key demographic and anthropometric characteristics. Exclusion criteria encompassed pregnant and lactating women; individuals with a history of alcohol consumption or smoking; those who had not adhered to a weight loss diet within the past 6 months; individuals taking nutritional supplements containing multivitamins or minerals; patients with chronic conditions, including hepatitis, autoimmune disorders and hepatic, renal, or cardiovascular diseases; non‐response to more than 40 food items in the food frequency questionnaire (FFQ), and energy intake less than 800 kcal or more than 4200 kcal.

All study participants signed a written consent form with explanations regarding the voluntary nature of participation in the project at baseline. Data collection was carried out using standard questionnaires and through face‐to‐face interviews by the researcher. General information was collected on age, education level, marital status and medical history of the individual, including disease. Anthropometric measurements were obtained according to World Health Organization (WHO) standards. Height was measured in centimetres using a non‐reversible tape measure with an accuracy of 0.5 cm attached to the wall, standing from the soles of the feet to the top of the head, without shoes, with the feet together and the heels together. Weight in kilograms was measured using an Omron digital scale, model HN286, with an accuracy of ±0.01 kg, standing, without shoes and with minimal clothing. Waist circumference was measured in centimetres with light clothing at the narrowest part between the iliac crest and the last rib using a non‐reversible tape measure by the researcher at the beginning of the study. Body mass index (BMI) was calculated by dividing the patients' weight in kilograms by the square of their height in meters squared.

Serum C‐reactive protein (CRP) levels were measured using a high‐sensitivity enzyme‐linked immunosorbent assay (hs‐ELISA) in a certified clinical laboratory. All samples were processed under standardised conditions, and internal quality control procedures included duplicate measurements and use of reference control samples. The reference range for CRP was < 5 mg/L, with values above this threshold considered elevated.

### 2.1 Dietary Data

The usual dietary intake of individuals in the year before the diagnosis of fatty liver for the case groups and before the interview for the control group was determined using a validated FFQ, which includes 147 food items. In this questionnaire, which includes a list of foods consumed by the Iranian population, participants were asked to report the frequency of consumption of each food item according to the standard size determined based on the option of times per day, week, month, or year. The obtained amounts of food items were calculated using the Household Scales Handbook converted to grams per day. Energy, macronutrient, and micronutrient intake were assessed using Nutritionist 4 software (First Databnk Inc., Hearst Cor., San Bruno, CA, USA).

### 2.2 The Dietary Inflammatory Index

The Dietary Inflammatory Index (DII) is a literature‐derived scoring algorithm developed to quantify the inflammatory potential of an individual's diet. It is based on a comprehensive review of 1943 peer‐reviewed articles published between 1950 and 2010, which examined the effects of various dietary components on six inflammatory biomarkers: CRP, IL‐1β, IL‐4, IL‐6, IL‐10 and TNF‐α. Each dietary parameter was assigned an inflammatory effect score (+1 for pro‐inflammatory, −1 for anti‐inflammatory and 0 for neutral).

In 2014, Shivappa et al. standardised the DII using data from 11 international dietary intake databases, creating a global reference database for 45 food parameters including nutrients, whole foods and bioactive compounds such as flavonoids, spices and tea. To calculate individual DII scores, dietary intake data obtained from a validated food frequency questionnaire (FFQ) were first energy‐adjusted using the residual method. Then, for each parameter, a *Z*‐score was calculated by subtracting the global mean intake from the participant's reported intake and dividing by the global standard deviation. These *Z*‐scores were converted to centred percentile scores and multiplied by the respective inflammatory effect scores. The final DII score was obtained by summing the weighted scores across all available parameters.

In this study, 36 dietary components were used to compute the DII, including: energy, protein, total fat, saturated fat, monounsaturated fat, trans fat, omega‐3 and omega‐6 polyunsaturated fats, cholesterol, carbohydrates, fibre, caffeine, vitamin A, beta‐carotene, thiamine, riboflavin, niacin, vitamin B6, folate, vitamin B12, vitamin C, vitamin D, vitamin E, iron, magnesium, selenium, zinc, garlic, onion, flavan‐3‐ols, flavones, flavonols, flavanones, anthocyanidins and isoflavones. A higher DII score indicates a more pro‐inflammatory diet, while a lower (more negative) score reflects a more anti‐inflammatory dietary pattern.

### 2.3 Statistical Analysis

In this study, statistical analysis of data was performed by SPSS software (Chicago LL version 26; SPSS Inc) and *p*‐value < 0.05 was considered statistically significant. Prior to statistical analysis, all data were reviewed for completeness and quality. Outlier detection was performed using boxplots and standardised *z*‐scores. Implausible values were verified against original records and excluded when necessary. Data cleaning included checking for consistency across variables and ensuring valid ranges. Missing data were minimal (< 5%) and handled using pairwise deletion for descriptive statistics and listwise deletion for regression analyses. These procedures were applied to maintain the integrity and reliability of the dataset and ensure robust statistical outcomes. The normality of the data was assessed using the Shapiro–Wilk test and visual inspection of histograms. Homogeneity of variances was evaluated using Levene's test prior to conducting parametric group comparisons. These tests confirmed that the assumptions for applying parametric statistical methods were adequately met.

Quantitative variables in the form of mean and standard deviation and qualitative variables in the form of number and percentages were reported. In order to compare qualitative and quantitative variables between the two groups, independent *t*‐test and chi‐square test were used, respectively. To examine the association between inflammatory indices and NAFLD status, binary logistic regression models were used. The two main independent variables were serum C‐reactive protein (CRP) and the Dietary Inflammatory Index (DII), each categorised into two groups based on clinically meaningful cutoffs. CRP was dichotomized using a threshold of 5 mg/L, and DII was split at the median value. Three models were constructed for each variable: Model 1: Crude (unadjusted), Model 2: Adjusted for age, Model 3: Fully adjusted for age, sex, BMI, marital status and education level. Odds ratios (ORs) and 95% confidence intervals (CIs) were reported.

### 2.4 Ethical Considerations

This research was conducted as a Master's thesis in Nutritional Sciences with the approval of the Research Council and Ethics Committee of the Faculty of Nutrition, Islamic Azad University, Science and Research Branch, Tehran, Iran (Code IR.IAU.SRB.REC.1403.085). The written informed consent was obtained from all individuals at the beginning of the study. If they did not wish to participate, they were allowed to withdraw from the project at any time. All information obtained from individuals remained completely confidential and was used solely for research purposes, and the patient's identity was kept confidential.

## 3 Results

Table 1 shows the demographic characteristics of the participants in the two study groups. The mean age of the NAFLD group was 47.7 ± 12.6 years and in Group Control 43.0 ± 14.1 years (*p* = 0.1). The body mass index (BMI) in the NAFLD group was 33.1 ± 2.7 kg/m^2^ and in the control group was 34.5 ± 4.8 kg/m^2^ (*p* = 0.1). No significant difference was found regarding demographic and anthropometric indices between the groups.

**TABLE 1:** Demographic characteristics of individuals in the case and control groups.

Variables		Case group (*n* = 42)	Control group (*n* = 43)	*p*
Age		47.7 ± 12.6	43 ± 14.1	0.1
Weight		89.1 ± 13.4	92 ± 15.4	0.3
Body mass index		33.1 ± 2.7	34.5 ± 4.8	0.1
Waist circumference		110 ± 8.9	109 ± 9.6	0.6
Waist‐to‐hip ratio		0.9 ± 0.04	0.9 ± 0.02	0.06
Education	Undergraduate	32 (76.7%)	32 (74.41%)	0.99
	Above the diploma	10 (23.3%)	11 (25.59%)	
Marriage	Single	14%	23.3%	0.2
	Married	86%	76.7%	
Gender	Man	55.8%	53.5%	0.8
	Woman	44.2%	46.5%	

Table 2 shows the mean values of nutritional intakes in the two NAFLD and control groups. The mean energy intake in the NAFLD group was 4719.3 ± 874 kcal/day and in the control group was 4709.5 ± 839.5 kcal/day (*p* = 0.9). No significant difference was found regarding macronutrient intake between the groups.

**TABLE 2:** Comparison of daily nutrient intake between obese individuals with and without fatty liver.

Nutrients	Case group (*n* = 42)	Control group (*n* = 43)	*p*
Energy (kcal)	4719.3 ± 874	4709.5 ± 839.5	0.9
Protein (g)	145.1 ± 25.4	145.5 ± 31.7	0.9
Carbohydrates (g)	817.2 ± 179.8	804.4 ± 162	0.7
Fat (g)	97.1 ± 12.8	101.2 ± 17.5	0.2
Saturated fatty acids (g)	28.7 ± 5.1	29.3 ± 6.7	0.6
Monounsaturated fatty acids (g)	26.3 ± 4.4	27.5 ± 6.3	0.3
Polyunsaturated fatty acids (g)	31.1 ± 4.1	33.0 ± 5.7	0.07
Selenium (mg)	0.48 ± 0.13	0.47 ± 0.11	0.5
Vitamin E (mg)	15.4 ± 3.03	15.2 ± 3.04	0.7
Vitamin A (μg)	2386.9 ± 764.9	2151.6 ± 749.2	0.1
Vitamin C (mg)	298.1 ± 87.8	304.1 ± 81.8	0.7
Vitamin D (μg)	2.8 ± 0.7	2.9 ± 1.4	0.6
Zinc (mg)	18.4 ± 3.3	18.2 ± 3.9	0.8
Iron (mg)	37.7 ± 8.3	37.2 ± 7.8	0.8
Copper (mg)	3.5 ± 0.7	3.4 ± 0.7	0.6
Trans fatty acids (g)	61.5 ± 18.3	60.7 ± 15.3	0.8
Fibre (g)	38.6 ± 6.4	37.6 ± 6.6	0.4
Thiamine (mg)	6.8 ± 1.7	6.7 ± 1.5	0.6
Calcium (mg)	1646.2 ± 208.4	1585.1 ± 284.3	0.2
Sodium (mg)	5597.5 ± 1248.9	5816.5 ± 1180.8	0.4
Sucrose (g)	26.0 ± 5.6	25.0 ± 5.0	0.4
Whole grains (g)	94.9 ± 32.0	97.4 ± 25.7	0.6
Refined grains (g)	2140.1 ± 678.1	2061.8 ± 546.4	0.5
Beans (g)	58.1 ± 22.1	59.3 ± 20.5	0.8
Brains (g)	4.7 ± 0.2	4.7 ± 0.4	0.9
Fruit (g)	639.5 ± 158.1	655.8 ± 116.8	0.5
Vegetables (g)	577.4 ± 187.3	523.7 ± 117.0	0.1
Dairy (g)	484.0 ± 111.0	466.4 ± 111.7	0.4

Table 3 presents the comparison of CRP and DII values between NAFLD and control groups. In the crude model, the mean CRP level was significantly higher in the NAFLD group (12.2 mg/L; 95% CI: 9.7–14.7) compared to the control group (8.5 mg/L; 95% CI: 6.0–11.0), with a *P*‐value of 0.04. After adjusting for age, the difference remained statistically significant (12.4 mg/L; 95% CI: 9.9–14.9 vs. 8.3 mg/L; 95% CI: 5.8–10.8; *p* = 0.02), indicating a consistent elevation of systemic inflammation in individuals with NAFLD. Regarding the Dietary Inflammatory Index (DII), no significant differences were observed between groups. In the crude model, the mean DII score was −4.0 (95% CI: −4.1 to −3.4) in the NAFLD group and−3.7 (95% CI: −4.1 to −3.4) in controls (*P* = 0.2). After age adjustment, the values remained similar (−4.0; 95% CI: −4.4 to −3.7 vs. –3.7; 95% CI: −4.0 to −3.4; *p* = 0.1), suggesting that dietary inflammatory potential may not differ significantly between groups.

**TABLE 3:** Comparison of outcome variables in the case and control groups.

Variables	Models	Case group	Control group	*p*
CRP	Crude model	12.2 (9.7–14.7)	8.5 (6–11)	0.04
	Model adjusted for age	12.4 (9.9–14.9)	8.3 (5.8–10.8)	0.02
DII	Crude model	−4 (−4.1, −3.4)	−3.7 (−4.1, −3.4)	0.2
	Model adjusted for age	−4 (−4.4, −3.7)	−3.7 (− 4, −3.4)	0.1

Logistic regression analysis was performed to assess the association between serum CRP and dietary inflammatory index (DII) with the presence of non‐alcoholic fatty liver disease (NAFLD) (Table 4). In the crude model, higher CRP levels were associated with increased odds of NAFLD (OR = 2.33; 95% CI: 0.9–5.5), though the association did not reach statistical significance. After adjusting for age, the association became significant (OR = 2.89; 95% CI: 1.1–7.2), indicating a positive relationship between systemic inflammation and NAFLD. In the fully adjusted Model 3, which included dietary intake and physical activity, the odds ratio increased to 4.21 (95% CI: 1.47–12.07), indicating a robust link between systemic inflammation and NAFLD.

**TABLE 4:** Association of CRP and DII with NAFLD using logistic regression models.

Variable	Model	Category 1 (Reference)	Category 2 (OR [95% CI])
CRP	Model 1	1	2.33 (0.9–5.5)
	Model 2	1	**2.89 (1.1–7.2)**
	Model 3	1	**4.21 (1.47–12.07)**
DII	Model 1	1	0.60 (0.2–1.4)
	Model 2	1	0.50 (0.2–1.2)
	Model 3	1	0.431 (0.17–1.11)

*Note:* Bolded values indicate statistically significant associations (*p* < 0.05). Model 1. Crude, Model 2. Adjusted for age, Model 3. Further adjustments for gender, BMI, marital status and education.

In contrast, DII was not significantly associated with NAFLD in either the crude model (OR = 0.60; 95% CI: 0.2–1.4) or the age‐adjusted model (OR = 0.50; 95% CI: 0.2–1.2), suggesting that dietary inflammatory potential may not independently predict NAFLD risk in this population. In the fully adjusted model, the inverse association between DII and fatty liver persisted but remained statistically non‐significant (OR: 0.431; 95% CI: 0.17–1.11). Although the trend suggests that a lower dietary inflammatory load may be protective, the lack of statistical significance implies that other factors may mediate the relationship between diet and liver health.

Also, no statistically significant linear association was found between Dietary Inflammatory Index (DII) and serum CRP levels (Model 1: *B* = −1.340, *p* = 0.092) (Table 5). Adjusting for potential confounders—including age, gender, BMI, marital status and education—did not substantially alter the results (Model 2: *B* = −1.240, *p* = 0.124; Model 3: *B* = −1.322, *p* = 0.113).

**TABLE 5:** Linear regression models predicting serum CRP based on DII and confounders.

Variable	Model 1 (*B*, *p*‐value)	Model 2 (*B*, *p*‐value)	Model 3 (*B*, *p*‐value)
DII score	−1.340, 0.092	−1.240, 0.124	−1.322, 0.113
Age	—	−0.050, 0.456	−0.067, 0.390
Gender	—	—	2.176, 0.282
BMI	—	—	−0.022, 0.927
Marital status	—	—	−0.120, 0.966
Education level	—	—	−0.881, 0.699

*Note:* Model 1 included only DII score; Model 2 adjusted for age; Model 3 adjusted for age, gender, BMI, marital status and education level.

## 4 Discussion

The present study was designed to investigate the association between serum inflammatory markers and dietary intake with fatty liver disease in obese individuals. In this study, serum CRP levels were significantly higher in the NAFLD group (12.2 mg/L) compared to the control group (8.5 mg/L), with both values exceeding the conventional reference range of < 5 mg/L [14]. This elevation indicates the presence of systemic inflammation in both groups, but the higher CRP in the NAFLD group suggests a more pronounced inflammatory state, potentially linked to hepatic involvement and disease progression. Elevated CRP levels have been consistently associated with metabolic dysfunction, insulin resistance and liver pathology in previous studies [15, 16]. For instance, CRP concentrations above 10 mg/L are considered indicative of acute or chronic inflammation and have been linked to increased risk of cardiovascular and hepatic complications [17]. Therefore, the observed difference, although modest, is clinically meaningful and supports the hypothesis that systemic inflammation plays a role in the pathophysiology of NAFLD. This finding is in line with the results of previous studies that have shown that chronic low–grade inflammation is considered one of the main axes in the development of steatohepatitis and progressive liver damage [18]. The increase in CRP in these patients may be due to the activation of macrophages and hepatic Kupffer cells in response to fat accumulation, insulin resistance and increased free fatty acids [19]. These processes may lead to increased production of inflammatory cytokines such as IL‐6 and TNF‐α, which ultimately stimulate CRP synthesis in the liver [20].

Regarding dietary‐induced inflammation, the Dietary Inflammatory Index (DII) did not differ significantly between the NAFLD and control groups. Therefore, our findings should be interpreted with caution. The absence of a significant association may reflect limited statistical power, measurement error inherent to FFQ‐based dietary assessment, or a true lack of relationship in this population. Nevertheless, systemic inflammation—reflected by elevated CRP levels—emerged as a distinguishing factor between obese individuals with and without NAFLD. Previous cohort and intervention studies have shown that diets with low inflammatory potential, such as the Mediterranean diet—rich in fruits, vegetables, whole grains, legumes, fish and olive oil—can inhibit or even partially reverse the progression of fatty liver [21, 22]. In contrast, pro‐inflammatory dietary patterns high in trans fats, added sugars and refined grains have been linked to worsening hepatic steatosis, insulin resistance and oxidative stress [23]. Several factors may explain the lack of significant DII differences in our study. First, the FFQ has inherent limitations in accurately capturing dietary intake and relies on participant recall. Second, the dietary composition of both groups may have been relatively similar in terms of macronutrients and anti‐inflammatory components included in the DII—such as fibre, antioxidant vitamins and flavonoids—especially given the widespread adoption of Western‐style diets in urban Iranian communities, characterised by high intake of saturated fats, simple sugars and processed foods.

Importantly, the absence of differences in energy and nutrient intake does not imply that diet plays no role in NAFLD development. One overlooked aspect may be the role of bioactive compounds such as polyphenols and flavonoids in modulating inflammation [24]. Although some flavonoid subclasses were included in the DII calculation, their impact may have been underestimated due to limited dietary sources or incomplete food composition data [25]. Future studies should consider direct measurement of polyphenol intake and serum metabolite levels to better elucidate their role in inflammation and liver health.

In this case–control study, we found no association between the DII and CRP levels in obese individuals with and without NAFLD. Furthermore, adjusting for potential confounders—including age, gender, BMI, marital status and education—did not substantially alter the results. Our findings are not fully aligned with previous studies that have reported a significant positive association between pro‐inflammatory diets and elevated CRP levels [26, 27]. One possible explanation for this discrepancy may be the relatively small sample size, which could have limited the statistical power to detect significant associations. Additionally, the observational nature of the study precludes any causal inference. It is also important to consider that CRP, while a widely used marker of systemic inflammation, can be influenced by various factors beyond diet, including genetics, physical activity, infections and metabolic status. Moreover, the DII, although validated, may not fully capture the complexity of dietary patterns or individual nutrient interactions in this specific population. Future studies with larger sample sizes, longitudinal designs and more comprehensive inflammatory markers may provide deeper insights into the role of diet‐induced inflammation in the pathogenesis of NAFLD.

Some strengths of this study include the selection of two groups with relatively homogeneous demographic and anthropometric characteristics, the accurate collection of dietary data using a validated questionnaire, and the use of the DII composite index as a validated measure of dietary inflammatory burden. However, this study has several limitations. First, its case–control design precludes causal inference. Second, the use of convenience sampling from two clinical centres may introduce selection bias and limit the generalizability of findings to broader populations. Third, dietary intake was self‐reported, which may be subject to recall bias and misreporting. Although the reported mean energy intake exceeded 4700 kcal/day, this level is consistent with the obesity status of participants in both groups (mean BMI > 33), and may reflect their habitual dietary patterns. Therefore, the high energy intake appears plausible and supports the internal validity of the dietary data. This should be considered when interpreting associations between dietary intake and inflammatory markers. Fourth, inflammatory assessment was limited to CRP, which does not capture the full spectrum of inflammatory activity. Finally, due to the limited sample size, we did not perform sensitivity analyses in order to preserve statistical power. Future longitudinal and interventional studies incorporating a broader panel of biomarkers—such as IL‐6, TNF‐α and adiponectin—are needed to clarify the temporal relationship between dietary patterns, inflammatory status and NAFLD progression. Additionally, integrating multi‐omic approaches such as metabolomics and gut microbiome profiling may offer deeper insights into the mechanistic links between diet‐induced inflammation and hepatic outcomes. Another limitation is the reliance on ultrasound alone for NAFLD diagnosis without supporting biochemical markers or assessment of inter‐observer reliability. Although ultrasound is widely used and non‐invasive, its sensitivity may vary depending on operator expertise and patient characteristics. Future studies should incorporate standardised imaging protocols alongside liver enzyme measurements to enhance diagnostic accuracy.

## 5 Conclusion

The findings of this study emphasise that inflammation plays a key role in the pathophysiology of fatty liver and that serum CRP level may be a valuable biomarker for identifying at risk individuals. Although no significant difference was observed in DII, this does not necessarily negate the importance of diet in the prevention of NAFLD. It is recommended that future studies use longitudinal and interventional designs, as well as more direct measurements of nutritional biomarkers, to gain a more accurate understanding of the interaction between nutrition, inflammation and fatty liver.

## Author Contributions

F.T., L.P. and S.A.K. contributed to the conception, design, and final drafting of the manuscript. F.T. contributed to data collection. F.T., L.P. and S.A.K. developed the statistical design and analysis. F.T., L.P. and S.A.K. contributed to the primary drafting of the manuscript. F.T., L.P. and S.A.K. supervised the study. All authors approved the final version for submission.

## Funding

The present work is part of a MSc thesis that was approved by the Azad University, Tehran, Iran. This study was fully funded by Azad University, Tehran, Iran.

## Ethics Statement

The study protocol was approved by the Ethics Committee of Mashhad University of Medical Sciences and Sabzevar University of Medical Sciences (Ethics Code: IR.MEDSAB.REC.1100.023) and was conducted according to the Declaration of Helsinki.

## Consent

Undersigned informed consent forms were obtained from all patients before the enrolment.

## Conflicts of Interest

The authors declare no conflicts of interest.

## Data Availability

The data that support the findings of this study are available from the corresponding author upon reasonable request.
